# Altruism, personal benefit, and anxieties: a phenomenological study of healthy volunteers' experiences in a placebo‐controlled trial of duloxetine

**DOI:** 10.1002/hup.2543

**Published:** 2016-07-05

**Authors:** Isaac N. Kwakye, Matthew Garner, David S. Baldwin, Susan Bamford, Verity Pinkney, Felicity L. Bishop

**Affiliations:** ^1^ Psychology, Faculty of Social and Human SciencesUniversity of SouthamptonSouthamptonUK; ^2^ Clinical and Experimental Sciences, Faculty of MedicineUniversity of SouthamptonSouthamptonUK; ^3^ University Department of Psychiatry and Mental HealthUniversity of Cape TownCape TownSouth Africa

**Keywords:** placebo effect, qualitative research, healthy volunteers, psychopharmacology, mental health

## Abstract

**Objective:**

The objective of this study was to develop an in‐depth understanding of healthy volunteers' experiences of mental health trials.

**Methods:**

A qualitative study was nested within a healthy volunteer placebo‐controlled trial of duloxetine, a psychotropic drug used for treating patients with major depression and generalized anxiety disorder. Eight participants were interviewed, and data were analyzed using interpretative phenomenological analysis.

**Results:**

Interviewees described volunteering for the trial because they were interested in research, wanted the monetary incentive, wanted to help researchers, and wanted to be part of something. On entering the trial, participants considered the possible risks and described feeling anxious, excited, and determined; they had some clear expectations and some loosely held hopes about what would happen. During the trial, participants were curious about whether they were taking duloxetine or placebo, self‐monitored their bodies' reactions, and guessed which treatment they received. On being un‐blinded to treatment allocation after completing the trial, some participants' guesses were confirmed, but others were surprised, and a few were disappointed.

**Conclusions:**

Small changes to advertising/consent materials to reflect volunteers' motivations could improve recruitment rates to similar trials; “active” placebos might be particularly useful for maintaining blinding in healthy volunteer trials; and sensitive procedures are needed for un‐blinding participants to treatment allocation. © 2016 The Authors. *Human Psychopharmacology: Clinical and Experimental* published by John Wiley & Sons, Ltd.

## Introduction

Participants in clinical trials engage in active meaning‐making processes that may influence trial validity (White *et al*., 2012). Studies that explore participants' experiences in clinical trials can shed light on such processes and may suggest design improvements for future trials (Snowdon *et al*., 1997).

Identifying participants' reasons for taking part in trials can suggest means to improve recruitment rates and reduce attrition. Patients appear to take part in clinical trials in physical health settings for three main reasons: because they seek personal benefit, typically in the form of wanting to improve their condition and/or symptoms (Cassileth *et al*., 1982; Tolmie *et al*., 2004; Stone *et al*., 2005; Kaptchuk *et al*., 2009; Scott *et al*., 2011; Macphail *et al*., 2012); because they want to help others or contribute to the development of new treatments, that is, altruism (Bevan *et al*., 1993; Hudmon *et al*., 1996; Tolmie *et al*., 2004; Chen and Johnson, 2009; Scott *et al*., 2011); and/or because they are curious about their condition and/or its treatment (Hudmon *et al*., 1996; Tolmie *et al*., 2004; Scott *et al*., 2011). Recruiting trial participants can also be challenging in mental health settings (Mason *et al*., 2007; Furimsky *et al*., 2008; Howard *et al*., 2009; Patterson *et al*., 2010; Jorgensen *et al*., 2014). Enquiries into patients' views in depression studies suggest that altruism can motivate trial participation (Grant *et al*., 2009; Tallon *et al*., 2011) while concerns about the specific treatment being tested and misunderstandings about research procedures can deter participation (Barnes *et al*., 2012).

Exploring participants' experiences in trials can highlight design features that require particular attention during informed consent, to ensure trials are conducted to the highest ethical standards. Participants can misunderstand the fundamental purpose of trials as well as key processes like randomization, believing that investigators are acting in a therapeutic capacity to provide the treatment that is best for them; this is known as the “therapeutic misconception” (Appelbaum *et al*., 1987; Snowdon *et al*., 1997; Featherstone and Donovan, 1998). Participants can also hold false beliefs about placebos, for example, believing that placebos have no effects at all (Criscione *et al*., 2003; Pope *et al*., 2003; Bishop *et al*., 2012c), which is not surprising given that information leaflets typically provide minimal information about placebos (Bishop *et al*., 2012a). Interestingly, the use of placebos might make trials particularly attractive to some groups of patients: for example, the chance to have a placebo (rather than active medication) encouraged some patients with schizophrenia to participate in a trial (Hummer *et al*., 2003).

Finally, exploring participants' attempts to make sense of their experiences in trials can help researchers to understand the potential impact of these sense‐making processes on trial validity. In one study, some patients with osteoarthritis were reluctant to report improved pain symptoms on questionnaires in case they had been “tricked” by receiving a placebo and would thus look foolish (Scott *et al*., 2011; White *et al*., 2012). Patients can be so eager to know whether they are receiving a placebo or “real” treatment that they seek cues to help them identify their treatment (Kaptchuk *et al*., 2009). For example, in a placebo‐controlled acupuncture‐trial patients reportedly guessed which treatment they were receiving based on noticing health benefits during the trial and/or attending to their physical reactions during this invasive treatment (Bishop *et al*., 2012b; White *et al*., 2012). Furthermore, a minority of patients might adopt quite determined approaches to identifying which treatment they are taking, for example, sending samples off for analysis (Zifferblatt and Wilbur, 1978; Howard *et al*., 1982). Attempts by participants to make sense of their experiences might thus introduce additional bias into assessments of subjective symptoms and subvert efforts to conduct blinded trials.

Overall, understanding the experiences of participants can help identify risks to the ethical and scientific validity of trials. However, current evidence is predominantly based on studies of patients with physical illness. Placebo‐controlled trials are also necessary and commonly used in mental health research (Baldwin *et al*., 2003; Khan *et al*., 2005; Dunlop and Banja, 2009), but the subjective experiences of participants in such trials have received less attention. This study explores healthy volunteer participants' experiences of a placebo‐controlled trial of duloxetine, a psychotropic drug used in treating depressed and anxious patients. We were particularly interested in exploring participants' experiences of entering the trial and taking duloxetine or placebo.

## Method

### Design

A qualitative study was embedded in a randomized placebo‐controlled drug trial, that is, participants for the qualitative study were recruited from participants in the trial. Interpretative phenomenological analysis (IPA) was used for this qualitative study, as it is well suited to elucidating participants' subjective, personal experiences of a placebo‐controlled trial. IPA is interested in the subjective meaning people give to events in their lives and is concerned with understanding how participants make meaning of (i.e., interpret) their personal and social world (Smith and Osborn, 2008; Smith *et al*., 2009). It is not interested in generalizing findings, rather it seeks an illuminating description based on in‐depth analysis of a small number of individual homogeneous cases (Osborn and Smith, 1998, e.g. Flowers *et al*., 1998). Ethical approval was obtained from the host institution (Reference Number 5887).

### The randomized placebo‐controlled trial

The trial examined the extent to which duloxetine alters response to CO_2_ inhalation in healthy volunteers compared with a placebo pill. Forty healthy men and women aged 18 to 55 years were recruited from the local community. Before entry, participants were screened for medical and psychiatric history (exclusions included past or present anxiety disorder or other mental health problems – for details see [Bamford *et al*., 2015]). Participants were randomized to receive either duloxetine or placebo for 2 weeks, starting at 30 mg then increasing to 60 mg daily (after 3 days) if the participant reported no concerning adverse effects. Short‐term adverse effects associated with duloxetine include dizziness, fatigue, nausea, constipation, dry mouth, diarrhea, headache, insomnia, and drowsiness. After 2 weeks, participants attended a single 3‐h testing session that evaluated the effects of duloxetine on emotional face processing (Bamford *et al*., 2015) and responses to a 7.5% CO_2_ inhalation challenge. Participants each received £100 for taking part.

### Qualitative sampling

All 17 participants who completed the trial during the qualitative study period (May – August 2013) were informed about this study and invited to participate. Eight agreed and were interviewed. Participants were four men and four women, aged from 20 to 44 years; six were students, and two were staff at the host institution. Five received placebo, and three received duloxetine. In accordance with IPA guidelines, the sample is homogeneous (in that all interviewees had participated in the same trial).

### Qualitative interviews

All participants provided *a priori* written‐informed consent before taking part in audio‐recorded phenomenological interviews by telephone (*n* = 7) or face‐to‐face (*n* = 1) (participants' choice). Interviews lasted for approximately 15 to 30 min and focused on the 2‐week drug administration phase of the trial, rather than the subsequent laboratory‐based testing session. A semi‐structured topic guide comprised 11 open‐ended questions that asked participants to describe their experiences, thoughts and feelings related to the trial (Appendix). This topic guide was used flexibly, allowing participants to control the direction of the interview. The interviews were conducted by INK under supervision from FLB, an experienced qualitative researcher. INK transcribed the interviews verbatim, anonymising identifiable details and giving participants pseudonyms.

### Analytic methods

The analytic approach aimed to describe and interpret the participants' phenomenological accounts (Willig, 2008) and followed published principles and guidelines for IPA (Smith *et al*., 1999). Before starting, the researcher (INK) attempted to put aside previous knowledge, experiences, and ideas he had about the phenomenon (Langdridge, 2007). The researcher then repeatedly read the transcripts to become familiar with the text, making notes in the left margin of anything that came to mind including comments, summaries, and associations. The analysis then proceeded in two phases: within‐participant and cross‐ participant. INK conducted the analysis with close supervision from FLB.

In the first (within‐participant) phase of the analysis, each transcript was analyzed separately. First, initial potential themes were identified based on the notes already made. These potential themes reflected psychological, phenomenological, and theoretical concepts and were recorded in the right hand margin of the transcript. Then, these potential themes were reviewed in the context of the original transcripts to identify and name clusters of themes for each participant, that is, low‐level themes (sub‐themes) that shared a common link or idea. Finally, a summary table for each participant was produced, comprising themes, sub‐themes, definitions, illustrative quotations, and line numbers from the original text.

In the second (cross‐participant) phase of the analysis, the researcher compared themes and sub‐themes across participants in order to identify common themes and new emerging ones. This involved comparing participants' summary tables and referring back with the original transcripts. A table of themes and constituent sub‐themes was generated and reviewed to identify a smaller number of superordinate themes, which reflect the essence of participants' experiences in the trial. This process was discussed in detail between the researcher (INK) and supervisor (FLB) and among the research team (co‐authors) to ensure the themes appropriately captured the essence of participants' accounts. Six superordinate themes were identified, two of which captured participants' experiences of the experimental lab testing session (“discomfort” and “credibility of the researchers”) and are not presented here. The remaining four superordinate themes focused on participants' experiences of the 2‐week drug trial and are presented in the Findings with illustrative quotes chosen for their clear articulation of the themes. Table 1 provides a summary of the superordinate and subthemes present in each participant's account. To protect their anonymity, participants are referred to by pseudonyms.

**Table 1:** Superordinate and subthemes across participants' accounts

	Christiana^a^	Mannix^b^	Rachael^b^	Kingsley^a^	Rosemond^b^	Roger^a^	Abigail^a^	Reuben^a^
Being attracted to the trial								
Participating for the money	✓			✓	✓		✓	✓
Wanting to learn	✓	✓	✓	✓	✓	✓	✓	✓
Wanting to help	✓				✓	✓		
Wanting to be a participant		✓	✓	✓		✓	✓	✓
Wanting to obtain health benefits		✓						
Anxieties on starting the trial								
Feeling anxious and excited	✓		✓	✓	✓		✓	
Determined to take part	✓		✓		✓			
Assessing the risks	✓				✓		✓	✓
Hopes and expectations about the drugs	✓			✓		✓	✓	
Curiosity about treatment allocation								
Knowing duloxetine has side effects	✓	✓	✓	✓	✓	✓	✓	✓
Placebos as sugar pills or sweets	✓	✓						✓
Placebos as not duloxetine	✓		✓		✓			
Placebos have psychological effects	✓	✓	✓	✓	✓	✓	✓	✓
Placebo as a control group				✓				
Educated guesses about treatment allocation	✓	✓	✓	✓	✓	✓	✓	✓
Uncertainty about physical changes		✓		✓		✓	✓	
Curious about which treatment they received	✓		✓	✓	✓		✓	✓
Finding out about treatment allocation								
Surprise! (or not…)	✓	✓	✓			✓	✓	✓
Feeling disappointed	✓						✓	
Making an important contribution	✓	✓		✓			✓	

a Participant was taking placebo.

b Participant was taking duloxetine.

## Findings

### Being attracted to the trial

The participants typically came across the study on the university webpage and found the £100 payment for participation very attractive. They thought the study sounded like an easy way to earn such an amount of money quickly. For example, Kingsley, who was a student, said his first reason for taking part was “the money” and was even happier to have been paid after he found out he had been taking the placebo: “It made me kind of happy that I had been erm given £ 100 to take like a sugar pills for 2 weeks.”

All of the participants who were attracted by the payment also talked about other attractive features; in particular, participants thought the trial would satisfy a desire to learn, to help, and/or to participate. For example, Rosemond wanted to learn about what goes on during randomized‐controlled trials and wanted to help, linking these desires to her own occupation: “Because I am a research student myself, so I understand how important is to help other people out sometime.” Rachael (another PhD student) wanted to be a participant and experience how it feels to be in a controlled trial.

Because I have never had experience in involving a trial, I thought it would be good opportunity to see what it is like to be a participant, just be on the other side to kind of help me when it come erm the experiencing how it feels to be a participant and what's the process involved. So that's the main motivation why I wanted to take part.

Mannix was the only participant who described being attracted to the trial because he thought it might be able to help him with his mild anxiety problems. He wanted to try the drug (duloxetine) to see if it would help with his symptoms:

Erm in the past I have struggled to, I had like problems with relaxing in, very stressing and anxious. So I thought it will be quite interesting to be involved in something that might be of help with that.

### Anxieties on starting the trial

Participants described having many thoughts running through their minds on starting the trial, including feelings of anxiety, excitement, and determination to take part. Some were both excited and anxious at the prospect of undertaking something new, as they did not know what to expect from being in a trial. Others, like Abigail, were very keen to take part but worried they may not meet the medical inclusion criteria.

A few of my friends had tried and they had been screened out of it, so I thought that my chances of actually being accepted onto the trial were quite slim.

Some participants tried to figure out if the study involved any risks of potential harm to their health. They were aware that they might experience side effects from the drug and talked about being confident that such effects would not have any major health implications for them. For example, Rosemond believed that the study must have been carefully scrutinized for safety before being allowed to proceed.

I knew that to have been accepted as a study then it must have gone through rigorous ethical procedures and everything must be licensed.

Rosemond's understanding of clinical research regulation thus gave her confidence that she would not be putting her health at significant risk by taking part.

All of the participants understood that taking part in the trial meant they would be given either duloxetine or placebo and some expressed no preference for one option over the other. Others saw participating in the trial as an opportunity to learn about their bodies and wanted to receive duloxetine so that they could see how their bodies would react to it. For example, Christiana hoped to take duloxetine right from the initial stages of the trial:

I suppose I, you hope that you will be taking the real erm oh I did that I shall be taking the real medication and then perhaps notice a difference of something but actually it wasn't, oh gosh it matters to me that much otherwise I should not have taken part but I think it was erm, perhaps just the feelings of oh I hope it's the real thing.

However, as for other participants, Christiana's hope to receive duloxetine was not so strong that it determined her participation in the trial: “I was keen even if I was going to take the placebo, I wanted to volunteer.”

### Curiosity about treatment allocation

Participants were typically quite curious about whether they were taking duloxetine or placebo. Their beliefs about duloxetine and placebo provided the context for this curiosity and their attempts to relieve it.

All the participants explained that placebos can have psychological effects, such as feeling hope and confidence. Rosemond was atypical in that her understanding of placebos incorporated both psychological and physiological effects:

If you imagine that you are taking erm taking an active drug erm yea I believe it could have psychological effects but also probably induce erm physiological effects to a certain degree which are brought about from psychological effect.

However, participants did not integrate their understanding of psychological placebo effects with their attempts to determine which treatment they were taking. Instead, they focused on the contrasting side effect profiles of duloxetine and placebo. Participants knew that duloxetine could trigger physical side effects (e.g., headaches), and this contrasted sharply with how they described placebos as harmless “sugar pills,” “sweets,” “non‐active drug‐substitutes,” and/or “controls.” They thus attended to their bodies and monitored themselves for side effects to inform their guesses as to whether they were taking duloxetine or placebo.

Some participants felt that their treatment allocation was obvious to them from very early on in the trial. Those who guessed that they were taking placebo had monitored their physical sensations and noticed that they had not experienced any symptoms; this made them think that they were taking placebo. For example, Christiana said:

I thought I hope erm I have experienced no side effects. I haven't felt different at all and I was beginning to think am sure I must be on placebo.

In comparison, those who guessed they were taking duloxetine did so because they had experienced physical changes, which they interpreted as side effects of duloxetine. For example, Mannix experienced headaches and other minor symptoms, which he attributed to duloxetine:

Every time I have a headache or something like that I assumed [it was] because I was on the duloxetine.

Some participants experienced more uncertainty and had doubts about what they were taking. These participants were unsure about how to interpret subtle physical changes, for example Kingsley described his uncertainty thus:

Erm there was a few occasions where I thought maybe I am reacting erm a little bit differently to what may be I would normally do. So I thought oh may be I am on the, ah may be am not on the placebo may be I have got the actual drug.

Whether participants were confident in making educated guesses or expressed doubts and were unsure, they were curious and wanted to be told for certain, by the researchers, what they had been taking.

### Finding out about treatment allocation

After the study, participants were informed by the senior researcher which treatment they had been allocated to, and they responded in various ways. Some were not surprised to be told they had been taking placebos, because this news was consistent with their educated guesses based on not experiencing any side effects during the trial. Others were not surprised to be told they had been taking duloxetine, because they had been experiencing side effects and (rarely) positive effects during the trial.

I think it was no surprise to me […] when I was told to ring back the next day and the erm professor told me, the senior lecturer or whatever that I was on placebo. (Christiana)

I wasn't too surprised when they told me I was on it [duloxetine] because like I said I felt quite relaxed and not very stressed about the work I have been doing and because of the headache and things like that. It wasn't a surprise. (Mannix)

In comparison, Rachael had not experienced side effects and was surprised to find out she had been taking duloxetine:

I tolerate the tablet very well that I didn't have any problems or any side effect. Erm I thought that I was convinced I was taking a placebo. I was quite surprised to hear when they said I was on the duloxetine.

Roger was surprised to be told he was taking placebo, because he had experienced a boost in his energy levels, which had made him think he was taking duloxetine. He made sense of this by drawing on his conceptualisation of psychological placebo effects as giving false confidence; he thought that this false confidence could in turn have increased his adrenaline levels and subsequently increased his energy levels.

Other participants who had been taking placebo also described more complex reactions, beyond simply being surprised or not. Some participants were disappointed to have been taking placebo because they doubted whether they had made an important contribution to the research. For example, Christiana said, “it was a bit of disappointment,” before going on to rationalize her feelings about her own contribution to the trial.

But then I tell myself rationally that they need people on placebo, they need people on the real medication. You you signed up [it was] very clearly explained in the information sheet, don't be so silly.

## Discussion

Four themes captured healthy participants' experiences in the 2‐week drug phase of a trial comparing duloxetine with placebo in an experimental medicine model of generalized anxiety disorder. These themes focused on enrolling in the trial, anxieties on commencing the trial, curiosity about duloxetine and placebo, and reactions on finding out which treatment they had been taking.

Participants enrolled in the trial not only for altruistic reasons but also to obtain a range of personal benefits, for example, money, knowledge, the experience of being a participant, and positive changes or improvements in their health. Previous studies in mental health have emphasized the role of altruistic motivations for taking part (Grant *et al*., 2009; Tallon *et al*., 2011). Our findings are more similar to (i) studies in physical health, which describe a wider range of motivations and (ii) a review of healthy volunteers' motivations, which found that while financial reward is the primary motivation participants also have other reasons for volunteering, including altruism, curiosity, interest in the research topic, and the potential for personal health benefits (Stunkel and Grady, 2011). Future trials in healthy volunteers might be able to enhance recruitment rates by identifying and emphasizing the diverse possible benefits to individuals of taking part. While emphasizing financial benefits in trial recruitment materials could be regarded as unethical coercion, emphasizing other benefits such as knowledge and understanding would be more appropriate.

At the start of the trial, participants had some anxieties about whether or not they would meet the eligibility criteria but were confident that the trial would not pose them any health risks despite being aware of the side effect profile of duloxetine. Researchers could consider whether participants' anxieties about eligibility might impact any measures of state anxiety taken during screening processes for specific clinical trials. A general understanding of the regulation of clinical trials seemed to foster confidence in the safety of this trial and could be explained during informed consent procedures to reassure others.

Participants were curious about whether they were taking duloxetine or placebo and made guesses about this, which were primarily based on self‐monitoring for duloxetine's side effects as described on the participant information sheet. A study of patients' experiences across various placebo‐controlled pharmacological trials found patients self‐monitored for positive and negative effects and attended to cues from doctors and laboratory tests in trying to understand whether or not they were receiving the placebo (Stone *et al*., 2005). It may be that healthy volunteers are more likely to self‐monitor for adverse effects of a drug because they do not have the same focus on symptoms that patients in a treatment trial seem to have. Trials in healthy volunteers might thus consider either explicitly stating in the information sheet that the placebo group might experience adverse effects and/or using active placebos (which mimic the side effect profile of the drug being tested) to help maintain blinding.

All of our participants wanted the researchers to tell them whether they had been taking duloxetine or placebo. Others have also reported that trial participants want to be un‐blinded at the end of a study so that they know which treatment they were allocated to (Di Blasi *et al*., 2005; Dinnett *et al*., 2005). While the majority of pregnant women from the “ORACLE” trial did not want to receive a summary of the trial results, those who were interested wanted information about their own personal treatment in the trial rather than a general summary of the results (Dixon‐Woods *et al*., 2006). The researchers interpreted this as women wanting to complete their own “personal narratives” around their experiences in relation to trial both during and after pregnancy. In a similar way, our findings can be interpreted as evidence that trial participants wanted to validate their own experiences in the trial, to confirm or inform their interpretations and thus develop more comprehensive personal accounts.

On being told their treatment allocation most of the participants were not surprised, as the news confirmed what they had already guessed. A few were surprised while others were disappointed and slightly worried that by taking placebo they might not have made a valuable contribution to the trial. Similarly, the majority of participants in a placebo‐controlled trial of corticosteroid for heel pain wanted to know which treatment they received and their reactions to being un‐blinded included surprise, excitement, and embarrassment (Di Blasi *et al*., 2005). Participants in a placebo‐controlled trial of acupuncture for irritable bowel syndrome also described emotional reactions (but not distress) to being un‐blinded, and some had to revise their interpretations of their experience in the trial in order to accommodate the surprising news that they had received placebo acupuncture (Bishop *et al*., 2012b). Despite the evidence that trial participants often want to be un‐blinded to treatment allocation (Di Blasi *et al*., 2005; Dinnett *et al*., 2005) and the contention that researchers are ethically obliged to un‐blind participants (Shalowitz and Miller, 2008), this is not common practice (Di Blasi, 2002). Our findings extend the existing literature on un‐blinding to treatment allocation by demonstrating that this is also important to healthy volunteers and that here, as in trials with patients, un‐blinding needs to be handled sensitively to address participants' concerns and help them make sense of their experiences.

This study has used IPA to provide an in‐depth analysis of healthy‐volunteer participants' experiences in one placebo‐controlled trial of duloxetine. As such, the findings are not intended to be directly generalized to other settings, but the insights gleaned may still have wider implications for the conduct of similar trials in future. The participants in this qualitative sub‐study were all members of an academic institution (as staff or students), even though the main trial recruited more widely from the community; future studies should explore the experiences of a more diverse range of participants.

## Conclusions

Healthy volunteer participants' accounts of their experiences in a placebo‐controlled trial of duloxetine focused on four main aspects: enrolling in the trial, anxieties on commencing the trial, curiosity about duloxetine and placebo, and reactions on finding out which treatment they had been taking. Participants wanted to take part in the trial for both personal gain and to help the researchers; they were concerned about their own eligibility and ability to contribute; they self‐monitored their health to help them to guess which treatment they were receiving; and being un‐blinded to treatment allocation helped them to make sense of their experiences. Practical suggestions arising from our findings include: small changes to advertising and/or informed consent materials to reflect volunteers' motivations may improve recruitment rates to similar trials; active placebos might be particularly useful for maintaining blinding in healthy volunteer trials; and it is important to use sensitive procedures for un‐blinding participants to treatment allocation, even when those participants are healthy volunteers rather than patients. Future studies should explore the experiences of healthy volunteers and patients in other mental health trials.

## Funding

The authors disclosed receipt of the following financial support for the research, authorship, and/or publication of this article. This research was funded by MRC grant MR/J011754/ awarded to M.G., D.S.B. and Marcus R. Munafo and which employed S.B. V.P. was funded by an ESRC PhD‐studentship. I.N.K. was funded by a Commonwealth Scholarship.

## Conflict of Interests

The authors declare that there is no conflict of interest.

## Ethics

The authors assert that all procedures contributing to this work comply with the ethical standards of the relevant national and institutional committees on human experimentation and with the Helsinki Declaration of 1975, as revised in 2008.

## Appendix Appendix Semi‐structured Interview Topic Guide

1. I am really interested in finding out about how you came to participate in the trial, and what was like for you. Please could you tell me all about it? [Prompt: I am interested in why you agreed to take part in the trial.]
2. What were your initial expectations and feelings?
3. As you know, being in this study involved you taking some tablets. At the start of the study, the researchers explained that some people would obtain duloxetine and some people would obtain placebo pills. Can you tell me how you felt about this at the start of the study?
4. And how about during the study, when you were taking the pills: did you have any thoughts about whether they were duloxetine or placebo?
  - Did you think you received real pills or placebo pills?
  - Why did you think so? What were the clues?
5. You should have been told at the end of the trial which pills you were taking. Can you tell me what it was like to find out?
6. How did you feel when you found out you had the [duloxetine/placebo]?
7. Did it make you think differently about anything?
  - Did it change the way you thought about the trial at all?
8. Do you have any ideas about what a placebo is? Do you think a placebo can have any effect on you? How do you think placebos work?
9. Is there anything else you would like to tell me about the pills that you were taking in the trial?
10. What, if anything, do you suggest researchers should do differently during the trial process?
11. Is there anything else you would like to say about taking part in the trial?
